# EXODUS: Stable and efficient training of spiking neural networks

**DOI:** 10.3389/fnins.2023.1110444

**Published:** 2023-02-08

**Authors:** Felix C. Bauer, Gregor Lenz, Saeid Haghighatshoar, Sadique Sheik

**Affiliations:** SynSense AG, Zurich, Switzerland

**Keywords:** spiking neural network (SNN), backpropagation (BP) algorithm, supervised learning, neuromorphic computing, neuromorphic algorithms, learning algorithm, computational efficiency, neuromorphic engineering

## Abstract

**Introduction:**

Spiking Neural Networks (SNNs) are gaining significant traction in machine learning tasks where energy-efficiency is of utmost importance. Training such networks using the state-of-the-art back-propagation through time (BPTT) is, however, very time-consuming. Previous work employs an efficient GPU-accelerated backpropagation algorithm called SLAYER, which speeds up training considerably. SLAYER, however, does not take into account the neuron reset mechanism while computing the gradients, which we argue to be the source of numerical instability. To counteract this, SLAYER introduces a gradient scale hyper parameter across layers, which needs manual tuning.

**Methods:**

In this paper, we modify SLAYER and design an algorithm called EXODUS, that accounts for the neuron reset mechanism and applies the Implicit Function Theorem (IFT) to calculate the correct gradients (equivalent to those computed by BPTT). We furthermore eliminate the need for ad-hoc scaling of gradients, thus, reducing the training complexity tremendously.

**Results:**

We demonstrate, via computer simulations, that EXODUS is numerically stable and achieves comparable or better performance than SLAYER especially in various tasks with SNNs that rely on temporal features.

## 1. Introduction

Spiking Neural Networks (SNNs) are a class of biologically-inspired networks with single bit activations, fine-grained temporal resolution and highly sparse outputs. Their memory makes them especially suitable for sequence tasks and their sparse output promises extremely low power consumption, especially when combined with an event-based sensor and asynchronous hardware (Göltz et al., 1995; Davies et al., 2021). SNNs have thus garnered considerable attention for machine learning tasks that aim to achieve low power consumption and/or biological realism (Cao et al., 2015; Diehl and Cook, 2015; Roy et al., 2019; Panda et al., 2020; Comşa et al., 2021).

SNNs are notoriously difficult to train due to the highly non-linear neuron dynamics, extreme output quantization and potential internal state resets, even for relatively simple neuron models such as Integrate-and-Fire (IF) or Leaky-Integrate-and-Fire (LIF) (Burkitt, 2006; Gerstner, 2021). Different approaches and learning rules have thus emerged to train SNNs. Biologically-inspired local learning rules (Choe, 2013; Lee et al., 2018; Lobov et al., 2020) do not rely on a global error signal, but often fail to scale to larger architectures (Bartunov et al., 2018). Methods that work directly with spike timings to calculate gradients bypass the issue of non-differentiable spikes, but are typically limited to time-to-first spike encoding (Göltz et al., 1995; Bohte et al., 2000; Mostafa, 2017). A notable exception to this has been proposed by Wunderlich and Pehle (2021), where gradients of threshold crossings are determined in continuous-time on an event-by-event basis, which results in reduced memory footprint. A parallelized, GPU-based implementation has been proposed recently (Nowotny et al., 2022).

Fueled by the success of deep learning, surrogate gradient methods more recently have paved the way for flexible gradient-based optimization in SNNs with the aim to close the accuracy gap to ANN counterparts (Esser et al., 2016; Bellec et al., 2018; Neftci et al., 2019; Safa et al., 2021).

Surrogate gradient methods make use of a smoothed output activation (usually a function of internal state variables) during the backward pass to approximate the discontinuous activation. This is well-supported in modern deep learning frameworks and allows the direct application of back-propagation through time (BPTT) to train SNNs. This alone would make it feasible to train SNNs successfully were it not for the high temporal resolution needed in SNNs. Activation is typically very sparse across time because data from an event-based sensor has a native time resolution of micro-seconds. This makes it necessary to simulate input using a lot of time steps on today's von Neumann machines, which work in discrete time. A naive implementation of BPTT with *T* discrete-time steps and *N* fully connected neurons incurs a computational complexity of order *O*(*N*^2^*T*) and a memory overhead of *O*(*NT*) on such architectures (Mart́ın-Sánchez et al., 2022), which makes training SNNs tremendously slow and computationally expensive.

To alleviate this issue, Shrestha and Orchard (2018) propose SLAYER, an algorithm in which gradients are back-propagated across layers as usual, but they are computed jointly in time such that the complexity due to *sequential* back-propagation in time is fairly eliminated by highly parallelized and GPU-accelerated joint gradient computation. Mathematically speaking, SLAYER can be seen as gradient computation for a modified forward computation graph underlying the chain rule, in which all the time dynamics are contracted into a single node (see Section 2.3 for further details). However, this comes at the cost of creating a loop in the computation graph where the gradients cannot be back-propagated via chain rule. To solve this issue, SLAYER ignores a term, known as reset kernel, which models the effect of output spike generation on the internal neuron potential. In e-prop (Bellec et al., 2020), a learning algorithm for recurrent SNNs, the same term among others is omitted in the gradient computaion for reasons of biological plausibility.

As a result, SLAYER yields a considerable speed-up for training SNNs at the cost of the deviation of gradients from what would be computed by BPTT, as well as some numerical instability. SLAYER typically deals with this instability by tweaking a hyperparameter which scales the gradient magnitude. This needs considerable hand-tuning and scales unfavorably to deeper architectures and longer time sequences. For completeness, it is possible to reduce computational complexity of gradient compuation in SNNs in other specialized implementations by optimizing the forward call (Knight et al., 2021), computing sparse gradients (Perez-Nieves and Goodman, 2021) or taking advantage of CUDA graph replay.

In this paper we propose EXODUS (EXact computation Of Derivatives as Update to SLAYER), in which we address numerical issues induced by SLAYER and compute gradients equivalently to what BPTT computes, while at the same time achieving a significant speedup of one order of magnitude in comparison to a non-optimized implementation. We achieve this by applying the Implicit Function Theorem (IFT) in a similar approach as Blondel et al. (2021), hence resolving the loopy structure in each layer's computation graph. Examples of other work that make use of the IFT to find gradients in the context of machine learning include (Scellier and Bengio, 2017; Bai et al., 2019), which formulate optimization processes for deep learning models as fixed point problems, as well as above mentioned (Wunderlich and Pehle, 2021).

In summary, our contributions are as follows:

(i) We improve the SLAYER algorithm by taking into account the reset response of neurons during the backward pass.

(ii) We compute gradients that are equivalent to BPTT and can be back-propagated through each layer.

(iii) We eliminate the need for ad-hoc scaling of gradients, needed for solving the numerical instability of SLAYER, thus, reducing the training complexity tremendously.

(iv) We demonstrate, via numerical simulations, that EXODUS is robust to changes in gradient scaling and achieves comparable or better performance than SLAYER in various tasks using snn that rely on temporal features.

## 2. Preliminaries and background

### 2.1. Implicit Function Theorem

In many problems in statistics, mathematics, control theory, machine learning, etc. the state of a problem is represented in terms of a collection of variables. However, in many cases, these variables are correlated due to existence of constraints. Here, we are interested in a setting where one may have a collection of *m*+*n* variables and a set of *m* equations (equality constraints) connecting them together. Since there are *m* equations, one may hope to solve for *m* variables, at least locally, as a function of the remaining *n* variables. It is conventional to call the first *m* variables *dependent* and the remaining *n* variables *independent* as the latter may vary (at least locally) independently of one another while the values of the former depend on the specific choice of those *n* independent variables.

The Implicit Function Theorem (IFT) provides rigorous conditions under which this is possible and specifies when the *m* dependent variables are differentiable with respect to *n* independent ones.

Theorem 2.1 (Implicit Function Theorem). Let **ϕ**:ℝ^*n*^×ℝ^*m*^ → ℝ^*m*^ be a differentiable function, let $Z=\left\{\left(x,y\right)\in {\mathbb{R}}^{n}\times {\mathbb{R}}^{m}:\phi \left(x,y\right)=0\right\}$ be the zero-set of **ϕ**, and let $\left(x_{0},y_{0}\right)\in Z$ be an arbitrary point in Z. If the *m*×*m* matrix $\frac{\partial \phi }{\partial y}\left(x_{0},y_{0}\right)$ is non-singular, i.e., $\det\frac{\partial \phi }{\partial y}\left(x_{0},y_{0}\right)\neq 0$, then,

- there is an open neighborhood $N_{\text{x}}$ around ***x***_0_ and an open neighborhood $N_{\text{x}}$ around ***y***_0_ such that $\frac{\partial \phi }{\partial y}\left(x,y\right)$ is non-singular for all $\left(x,y\right)\in N:=N_{x}\times N_{y}$ [including of course the original point (***x***_0_, ***y***_0_)].
- there is a function $\psi :N_{x}\to N_{y}$ such that (***x***, **ψ**(***x***)) belongs to the zero-set Z, i.e., **ϕ**(***x***, **ψ**(***x***)) = 0, for all $x\in N_{x}$. Therefore, ***y*** = **ψ**(***x***) can be written as function of ***x***.
- (Chain rule) **ψ** is a differentiable function of ***x*** in $N_{x}$ and

(1) $$\begin{array}{l} \frac{\partial \phi }{\partial y}\cdot \frac{\partial \psi }{\partial x}+\frac{\partial \phi }{\partial x}=0, \end{array}$$

which from the non-singularity of $\frac{\partial \phi }{\partial y}$ yields


(2) $$\begin{array}{l} \frac{\partial \psi }{\partial x}=-\frac{\partial \phi }{\partial y}{}^{-1}\cdot \frac{\partial \phi }{\partial x}. \end{array}$$

Remark 2.2. Note that here, for simplicity, we denoted the independent and dependent variables with ***x***∈ℝ^*n*^ and ***y***∈ℝ^*m*^. In general, one may choose any disjoint subsets of the variables of size *m* and *n* as dependent and independent variables and verify the conditions of the IFT.

In Appendix 1, we provide examples to illustrate how IFT is applied for computing the derivative of the dependent variables with respect to independent ones. In particular, we show that intuitive ad-hoc application of the chain rule in a loopy computation graph and neglecting the conditions of ift may indeed yield wrong results.

### 2.2. Spike Response Model

Neuron dynamics in SNNs can be described by a state-space model where the internal state of each neuron depends on both its current input and its previous states. When applying the conventional back-propagation algorithm, therefore, the gradients need to be back-propagated not only through layers of the network but also through time. This paper builds upon SLAYER (Spike LAYer Error Reassignment) (Shrestha and Orchard, 2018), an algorithm for training feedforward SNNs. SLAYER is based on the spike response model (SRM) (Gerstner, 2021), where the state of a spiking neuron at each time instant *n* is described by its membrane potential *u*[*n*], given by


(3) $$\begin{array}{ll} u\left[n\right] & =\underset{i}{\sum }w_{i}\left(\epsilon *s_{i}^{in}\right)\left[n\right]+\left(\nu *s^{out}\right)\left[n-1\right], \end{array}$$

(4) $$\begin{array}{ll} s^{out}\left[n\right] & =f_{s}\left(u\left[n\right]\right). \end{array}$$

Here $s_{i}^{in}\left[n\right]$ and *w*_*i*_ denote the input spikes received from the *i*-th pre-synaptic neuron and the corresponding weight. Furthermore, **ϵ** and **ν** denote the spike response and reset kernel of the neuron (with * denoting the convolution operation in time) to the incoming and outgoing spikes, where a discrete delay of size 1 is introduced for the reset kernel **ν** to defer the effect of outgoing spikes to the next time instant.

Outgoing spikes are obtained from the membrane potential through a memory-less binary spike generating function *f*_*s*_:ℝ → {0, 1} where *f*_*s*_(*u*[*n*]) = 1 when the membrane potential *u*[*n*] reaches or exceeds the neuron firing threshold θ>0, and is 0 otherwise. Since *f*_*s*_ is not differentiable, a common solution for obtaining well-defined gradients for training SNNs is to use *f*_*s*_ in the forward pass to produce outgoing spikes but replace it with a differentiable function in the backward pass, where the gradients are computed through back-propagation. This is known as the surrogate gradient method in SNN literature. Different surrogate gradients have been proposed such as piecewise linear (Bohte, 2011; Esser et al., 2016; Bellec et al., 2018, 2020), tanh (Woźniak et al., 2020), fast sigmoids (Zenke and Ganguli, 2018), or exponential functions (Shrestha and Orchard, 2018). In this paper, with some abuse of notation, we denote the surrogate gradient by $f_{s}^{\prime }\left(.\right)$. Our derivations are valid for any $f_{s}^{\prime }$ as long as $f_{s}^{\prime }\left(u\left[n\right]\right)$ is well-defined for all feasible values of the membrane potential *u*[*n*] at all time instants *n*.

### 2.3. Vectorized network model

As in Shrestha and Orchard (2018), we focus on a feedforward network architecture with *L* layers. Using Equations (3, 4) and applying vectorization, we may write the forward dynamics of a layer *l* with *N*_*l*_ neurons and input weights $W^{\left(l-1\right)}\in {\mathbb{R}}^{N_{l}\times N_{l-1}}$ equivalently as:


(5) $$a^{\left(l\right)}[n]\;=W^{\left(l-1\right)}s^{\left(l-1\right)}[n],$$

(6) $$z^{\left(l\right)}[n]\;=(\text{\textbackslash{}}\epsilon *a^{\left(l\right)})[n],$$

(7) $$u^{\left(l\right)}[n]\;=z^{\left(l\right)}[n]+(\nu *s^{\left(l\right)})[n-1],$$

(8) $$\begin{array}{ll} {\text{s}}^{\left(l\right)}\left[n\right] & =f_{s}\left({\text{u}}^{\left(l\right)}\left[n\right]\right), \end{array}$$

Here, *a*^(*l*−1)^[*n*] represents the weighted input spikes (output spikes of previous layer) *s*^(*l*−1)^. Filtering/smoothing out by the neuron spike response **ϵ** yields the post-synaptic response **z**^(*l*)^.

We take {*s*^(*l*−1)^[*n*]:*n*∈[*T*]} and {*s*^(*l*)^[*n*]:*n*∈[*T*]} as the input and output of a specific layer *l*∈[*L*] across *T* time instants, where we used the short-hand notation [*N*] = {1, 2, …, *N*}. The model output is given by {*s*^(*L*)^[*n*]:*n*∈[*T*]}. We also define the loss as $L\left({\text{s}}^{\left(L\right)}\left[0\right],...,{\text{s}}^{\left(L\right)}\left[T-1\right]\right)$ in terms of the network output over all time instants [*T*].

**Figure 1:**
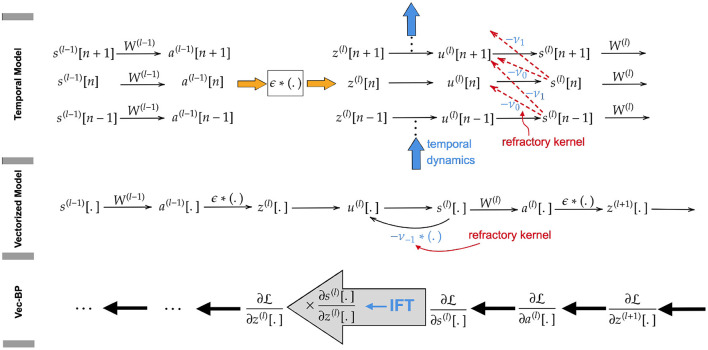
**(Upper)** Directed acyclic computation graph along temporal and spatial dimension. **(Middle)** Loopy computation graph after vectorization along the temporal dimension. The loop is introduced by the reset kernel and is ignored in the backward path of SLAYER. **(Lower)** Vectorized backward path, applying IFT to back-propagate the gradients through the loopy computation graph.

The computational graph of the described network model has a directed acyclic graph structure in spatio-temporal dimensions, as illustrated in Figure 1. Therefore, the gradients can be propagated from the loss L to the trainable weights backward across the layers (spatial) and also time instants (temporal). However, as explained above, this approach is computationally slow.

The SLAYER algorithm introduced by Shrestha and Orchard (2018) avoids this by vectorizing the variables over time, as illustrated in Figure 1. Instead of assigning to each state variable at each point in time an individual node in the computational graph, here every node corresponds to a state across all time instants. This, however, introduces loops in the computational graph due to the mutual dependence of the vectorized variables **u**^(*l*)^[.] and *s*^(*l*)^[.] in Equations (7, 8). This prohibits back-propagating through these variables. To solve this issue, SLAYER ignores the reset kernel **ν** [effect of output spikes on neuron potential in Equation (7)] in the calculation of its gradients. Apart from this omission, the algorithm is equivalent to ours, as described in detail below.

## 3. Derivation of EXODUS gradients

### 3.1. Vector back-propagation

We calculate gradients precisely by taking into account the reset kernel neglected by SLAYER. To do so, we apply the ift to back-propagate the gradients through the loopy computation graph. As the loops occur only between the variables **u**^*l*^[.] and **s**^(*l*)^[.] within the same layer, we apply IFT to each layer *l*∈[*L*] individually. More specifically, by applying the chain rule, we back-propagate the gradients from the last layer to compute $\frac{\partial L}{\partial {\text{s}}^{\left(l\right)}\left[.\right]}$. We show that, although there is a loop between **u**^(*l*)^[.] and **s**^(*l*)^[.], we are still able to compute $\frac{\partial {\text{s}}^{\left(l\right)}\left[.\right]}{\partial {\text{a}}^{\left(l\right)}\left[.\right]}$ (see Figure 1). Multiplying this gradient with $\frac{\partial L}{\partial {\text{s}}^{\left(l\right)}\left[.\right]}$ enables us to compute $\frac{\partial L}{\partial {\text{a}}^{\left(l\right)}\left[.\right]}$, which is the gradient back-propagated to the previous layer.

### 3.2. Derivations for a generic model

To apply IFT, we need to specify the underlying equations and also the dependent and independent variables. Let us consider (Equations 7, 8) for all *n*∈[*T*]:


$$\begin{array}{l} \phi_{u}^{\left(l\right)}[n]:=u^{\left(l\right)}[n]-z^{\left(l\right)}[n]-(\nu *s^{\left(l\right)})[n-1]=0,\;\\ \phi_{s}^{\left(l\right)}[n]:=s^{\left(l\right)}[n]-f_{s}(u^{\left(l\right)}[n])=0. \end{array}$$

This is a system of 2*N*_*l*_*T* equations in terms of three vectorized variables **u**^(*l*)^[.], **s**^(*l*)^[.], **z**^(*l*)^[.], each of dimension *N*_*l*_*T*. Therefore, by a simple dimensionality check, we can see that we may write two of these variables (dependent) as a differentiable function of the other variable (independent) provided that the conditions of IFT hold.

To pass the chain rule through the loopy computation graph at layer *l*, we need to compute $\frac{\partial {\text{s}}^{\left(l\right)}\left[.\right]}{\partial {\text{z}}^{\left(l\right)}\left[.\right]}$ (see, e.g., Figure 1). This implies that we need to treat **z**^(*l*)^[.] as the independent and (**s**^(*l*)^[.], **u**^(*l*)^[.]) as the dependent variables. For simplicity of notation, we denote these independent and dependent variables and the corresponding equations by


$$\begin{array}{l} x^{\left(l\right)}:=\{z^{\left(l\right)}[n]:n\in [T]\},\psi^{\left(l\right)}:=\{u^{\left(l\right)}[n],s^{\left(l\right)}[n]:n\in [T]\},\;\\ \phi^{\left(l\right)}:=\{\phi_{u}^{\left(l\right)}[n],\phi_{s}^{\left(l\right)}[n]:n\in [T]\}. \end{array}$$

Let ${\text{J}}^{\psi^{\left(l\right)}}=\frac{\partial \phi^{\left(l\right)}}{\partial \psi^{\left(l\right)}}\in {\mathbb{R}}^{2N_{l}T\times 2N_{l}T}$ and ${\text{J}}^{x^{\left(l\right)}}=\frac{\partial \phi^{\left(l\right)}}{\partial x^{\left(l\right)}}\in {\mathbb{R}}^{2N_{l}T\times N_{l}T}$ be the Jacobian matrices of the equations **ϕ**^(*l*)^ with respect to the dependent and independent variables, respectively. Let us also define ${\text{G}}^{\left(l\right)}=\frac{\partial \psi^{\left(l\right)}}{\partial x^{\left(l\right)}}\in {\mathbb{R}}^{2N_{l}T\times N_{l}T}$ as the gradients of dependent variables with respect to the independent ones.

With this, we verify the IFT conditions: (i) All the equations are differentiable, provided that *f*_*s*_ is a differentiable function. (ii) By bringing **J**^ψ^^(*l*)^ into a row-echelon form, we can prove that **J**^ψ^^(*l*)^ is non-singular. We refer to Appendix 2 for a detailed derivation. The gradients **G**^(*l*)^ are then found by solving IFT Equation (1) **J**^ψ^^(*l*)^·**G**^(*l*)^ = −**J**^*x*^^(*l*)^.

Applying a forward substitution method (see Appendix 2) yields the desired gradients:


(9) $$\begin{array}{ll} \sigma_{m}^{\left(l\right)}[n] & :={\left(\partial s^{\left(l\right)}[.]z^{\left(l\right)}[.]\right)}_{n,m}=\partial s^{\left(l\right)}[n]z^{\left(l\right)}[m]\;\\ & =\{\begin{array}{ll} 0 & n<m\\ f^{\prime (l)}[n] & n=m\\ f^{\prime (l)}[n](\nu *\sigma_{m}^{\left(l\right)})[n-1] & n>m, \end{array} \end{array}$$

where **f**^′(*l*)^[*n*] is the *diagonal matrix* holding the surrogate gradients ${\left({\text{f}}^{\prime \left(l\right)}\left[n\right]\right)}_{ii}=f_{s}^{\prime }\left(u_{i}^{\left(l\right)}\left[n\right]\right)$.

As we explained before, computing $\frac{\partial {\text{s}}^{\left(l\right)}\left[.\right]}{\partial {\text{z}}^{\left(l\right)}\left[.\right]}$ *via* IFT allows us to push the back-propagation (chain rule) through loops in the computational graph.

The remaining steps needed for back-propagation are quite straightforward and are obtained from Equations (5, 6) as follows:


(10) $$\begin{array}{l} \;\frac{\partial z^{\left(l\right)}[m]}{\partial z^{\left(l\right)}[n]}=\frac{\partial (\epsilon *a^{\left(l\right)})[m]}{a^{\left(l\right)}[n]}=\frac{\partial \sum_{k=1}^{m}\epsilon_{m-k}\cdot a^{\left(l\right)}[k]}{a^{\left(l\right)}[n]}=\epsilon_{m-n}\cdot I \end{array}$$

(11) $$\;\frac{\partial a^{\left(l\right)}[m]}{\partial s^{\left(l-1\right)}[n]}=\delta_{m,n}\cdot W^{\left(l-1\right)}$$

(12) $$\;\frac{\partial a^{\left(l\right)}[m]}{\partial W^{\left(l-1\right)}}=s^{\left(l-1\right)}{\left[m\right]}^{⊤},$$

where ***I*** denotes the identity matrix and ⊤ the transpose operation. Similar to Shrestha and Orchard (2018), we define **e**^(*l*)^[*n*] and **d**^(*l*)^[*n*] as the derivative of the loss L with respect to the spike output and the weighted input, respectively, of layer *l* at time *n*. Making use of Equations (10, 11) we obtain:


(13) $$\begin{array}{l} e^{\left(l\right)}[n]:=\frac{\partial ℒ}{\partial s^{\left(l\right)}[n]}=\sum_{m=n}^{T}\;\frac{\partial ℒ}{\partial [a^{\left(l+1\right)}[m]}\frac{\partial [a^{\left(l+1\right)}[m]}{\partial s^{\left(l\right)}[n]}=d^{\left(l+1\right)}[n]W^{\left(l\right)} \end{array}$$

(14) $$\begin{array}{l} d^{\left(l\right)}[n]:\;=\frac{\partial ℒ}{a^{\left(l\right)}[n]}=\sum_{m=n}^{T}\sum_{k=m}^{T}\frac{\partial ℒ}{\partial s^{\left(l\right)}[k]}\frac{\partial s^{\left(l\right)}[k]}{\partial s^{\left(l\right)}[k]z^{\left(l\right)}[m]}\frac{\partial z^{\left(l\right)}[m]}{\partial a^{\left(l\right)}[n]}\\ \;=\sum_{m=n}^{T}\sum_{k=m}^{T}e^{\left(l\right)}[k]\sigma_{m}^{\left(l\right)}[k]\epsilon_{m-n}. \end{array}$$

These equations are solved for **e**^(*l*)^[.] and ***d***^(*l*)^[.] iteratively starting from ${\text{e}}^{\left(L\right)}\left[.\right]=\frac{\partial L}{\partial {\text{s}}^{\left(L\right)}\left[.\right]}$ at the last layer. Using Equation (12), we arrive at the gradients of the loss with respect to weight matrix **W**^(*l*)^:


(15) $$\begin{array}{ll} \frac{\partial ℒ}{\partial W^{\left(l\right)}} & =\sum_{n=1}^{T}\frac{\partial ℒ}{\partial a^{\left(l+1\right)}[n]}\;\frac{\partial a^{\left(l+1\right)}[n]}{\partial W^{\left(l\right)}}\;=\sum_{n=1}^{T}d^{\left(l+1\right)}[n]\cdot s^{\left(l\right)}{\left[n\right]}^{⊤} \end{array}$$

### 3.3. Simplification for LIF and IF neurons

Our derivation of the gradients in the previous section applies to an arbitrary Spike Response Model (SRM). Since many SNNs are based on the Leaky Integrate-and-Fire (LIF) neuron model, in this section, we derive a more compact expression for this special case. Using the same notation as in Section 2.2, LIF dynamics can be expressed in terms of the SRM (Gerstner, 2021) by choosing spike response and reset kernels $\epsilon_{n}=\alpha^{n}1_{\left\{n\geq 0\right\}}$ and $\nu_{n}=-\alpha^{n}\theta 1_{\left\{n\geq 0\right\}}$, respectively. Here, $\alpha :=\exp\frac{-\Delta }{\tau }\in \left(0,1\right)$ is the decay factor in the LIF model determined by the membrane time constant τ and simulation time step Δ. Furthermore, 1 denotes the indicator function and θ the firing threshold. This analysis can be applied equivalently to if neurons without leak by setting the decay factor α≡1.

The derivatives of **s**^(*l*)^[.] in Equation (9) can be expressed in closed-form as follows


(16) $$\begin{array}{l} \begin{array}{l} \sigma_{m}^{\left(l\right)}[n]\;=\{\begin{array}{ll} 0 & n<m\\ f^{\prime (l)}[n] & n=m\\ -\theta f^{\prime (l)}[n]f^{\prime (l)}[m]\;\chi_{m}{}^{\left(l\right)}[n] & n>m \end{array} \end{array} \end{array}$$

(17) $$\chi^{\left(l\right)}{}_{m}[n]:=\{\begin{array}{ll} \text{I} & n=m+1\\ \prod_{k=m+1}^{n-1}\left(\alpha I-\theta f^{\prime (l)}[k]\right) & n>m+1 \end{array}$$

where ***I*** denotes the identity matrix. We refer to Appendix 3 for further details.

#### 3.3.1. Computational efficiency

In the following we show how the gradients can be computed efficiently for LIF and IF neurons. We first note that the gradient terms ***e***^(*l*)^ and $\frac{\partial L}{\partial W^{\left(l\right)}}$ (Equations 13, 14) are matrix products, for which efficient GPU-accelerated implementations are commonly available. The remaining complexity arises from the terms **d**^(*l*)^ (Equation 14), which can be simplified drastically for the given neuron dynamics. To this end, we introduce an auxiliary variable $\zeta_{n}^{\left(l\right)}\left[k\right]$, defined for *n* ∈ [*T*], *l* ∈ [*L*]and*k* ≥*n*, as


(18) $$\begin{array}{l} \zeta_{n}{{}^{\left(1\right)}}_{}[k]:=\{\begin{array}{ll} \;I & k=n\\ \left(\prod_{m=n}^{k-1}\alpha \text{I}-\theta {\text{f '}}^{\left(1\right)}[m]\right) & k>n. \end{array} \end{array}$$

As demonstrated in Appendix 3.1, for LIF and IF neurons, Equation 14 can be expressed as


(19) $$d^{\left(l\right)}[n]=\sum_{k=n}^{T}e^{\left(l\right)}[k]f^{\prime (l)}[k]\;\zeta^{\left(l\right)}{}_{n}[k],$$

which can be computed in O(T) time. Furthermore, the vector components of **d**^(*l*)^ are independent of each other, allowing for a highly parallelized, GPU-accelerated gradient computation. From this analysis we expect EXODUS to reach similar computational efficiency as SLAYER (Shrestha and Orchard, 2018). The experimental results in Section 4.5 show that this is indeed the case.

#### 3.4. Comparison with SLAYER gradients

By comparing the gradients computed in Section 3 with those in SLAYER (Shrestha and Orchard, 2018), we find that the expressions for *e*^(*l*)^[.] and $\frac{\partial L}{\partial {\text{W}}^{\left(l\right)}}$ (see Equations 13, 15) match. The difference lies mainly in **d**^(*l*)^ (Equation 14) and more specifically in the derivatives $\frac{\partial {\text{s}}^{\left(l\right)}\left[n\right]}{\partial {\text{z}}^{\left(l\right)}\left[m\right]}$, which are set to 0 for *m*≠*n* in SLAYER. In particular, we would obtain the gradients in SLAYER by setting the reset kernel to 0, in which case $\sigma_{m}^{\left(l\right)}\left[n\right]$ is only nonzero for *m* = *n*. In the concrete case of LIF neuron dynamics (cf. Section 3.3), we may indeed notice that the quality of the approximation of the gradients in SLAYER depends on how the matrix $\chi_{m}^{\left(l\right)}$ (see Equation 17) decays as a function of index-difference |*n*−*m*−1|. This decay, in general, can be characterized in terms of singular values of **f**^′(*l*)^[*n*]. However, since the matrix of surrogate gradients **f**^′(*l*)^[*n*] are all diagonal, this boils down to how the diagonal elements


$$\begin{array}{ll} \chi_{m}^{\left(l\right)}\left[n\right]{}_{i,i} & =\overset{n}{\underset{k=m+1}{\prod }}\left(\alpha -\theta f_{s}^{\prime }\left(u_{i}^{\left(l\right)}\left[k\right]\right)\right) \end{array}$$

decay as a function of *n*−*m*−1 (for *n*≥*m*+1). We may study several interesting scenarios:

(i) In the usual case where *f*_*s*_ is an increasing function, $f_{s}^{\prime }\left(u\right)$ is positive for all *u*∈[0, θ]. If in addition, one designs the surrogate gradient $f_{s}^{\prime }$ such that $f_{s}^{\prime }\left(u\right)\in \left[0,\frac{\alpha -\mu }{\theta }\right]$ for some μ∈[0, α] for all *u*∈[0, θ], one may obtain the bounds $0\leq \chi_{m}^{\left(l\right)}\left[n\right]{}_{i,i}\leq \mu^{n-m-1}$, which implies that $\chi_{m}^{\left(l\right)}\left[n\right]{}_{i,i}$ is quite small if $n\geq m+1+O\left(\log\frac{1}{\mu }\right)$. As a result, compared with SLAYER, which assumes $\chi_{m}^{\left(l\right)}\left[n\right]\equiv 0$ for all *m, n, l*, our method takes into account additional $O\left(\log\frac{1}{\mu }\right)$ correction terms.
(ii) If the surrogate gradient is not designed properly such that it is larger than $\frac{\alpha }{\theta }$ for some range of *u* within [0, θ], the term $\chi_{m}^{\left(l\right)}\left[n\right]$ may become significantly large as *n*≫*m*+1. In such a case, we may expect a large deviation between the corrected gradients derived from our method and the approximate gradients in SLAYER. We speculate that this may be the reason SLAYER gradients are more sensitive to the scaling of the surrogate gradients, as shown in our results.

## 4. Simulation results

We show that EXODUS leads to faster convergence than SLAYER when applied to different tasks, as our method computes the same gradients as BPTT. We benchmark both algorithms on four neuromorphic tasks with increasingly temporal features. To compare numerical stability, experiments are repeated over different gradient scaling factors, incorporated in the surrogate gradients $f_{s}^{\prime }$ (see Section 2.2). It is worthwhile to mention that in our experiments we do not aim to illustrate superior classification performance of our algorithm compared with other state-of-the-art algorithms, but the achievable performance improvement over SLAYER. We make a conscious decision not to use any training tricks that improve accuracy (learning rate schedulers, data augmentation, etc.) as this would harm a direct comparison. In brief, any results in literature that have been obtained by using BPTT can be recreated using EXODUS if the neuron model supports it, because we compute the same gradients (see Section 5 in the Appendix for a numerical demonstration). When comparing EXODUS and SLAYER, we make sure that forward pass neuron dynamics, random seed and initial weights are the same. We used SLAYER's official PyTorch implementation available on Github. Detailed architecture and training parameters are described in the Table 1 in Appendix.

### 4.1. CIFAR10-DVS

This dataset is a neuromorphic version of the original image classification dataset (Li et al., 2017). For this experiment we used a spiking ResNet Wide-7B architecture with 1.19M parameters (Fang et al., 2021). The best previously published result using that architecture achieves 70.2% accuracy for 8 time steps and LIF neurons, which we beat with both EXODUS and SLAYER (see Table 1). EXODUS achieves the highest accuracy overall at 72.28%.

**Table 1:** Validation accuracy and backward pass speedup per epoch for different datasets.

	**Validation accuracy [%]**		**Mean backward pass speedup compared to BPTT**	
	**EXODUS**	**SLAYER**	**EXODUS**	**SLAYER**
CIFAR10-DVS	**72.28 ± 0.13**	71.53 ± 0.18	–	–
DVS gesture (5 layers)	**92.8 ± 2.2**	87.8 ± 3.0	**16.57 × **	14.09 ×
DVS gesture (8 layers)	**94.54 ± 0.8**	93.64 ± 0.49	–	–
SHD	**78.01 ± 0.2**	70.58 ± 1.9	9.1 ×	**11.22 × **
SSC	**55.41 ± 0.4**	40.1 ± 0.8	**17.3 × **	14.59 ×
Average for 3 datasets			**14.3 × **	13.3 ×

Validation accuracy is mean and standard deviation of maximum accuracies across three runs with a gradient scale of 1. Backward pass speedup is measured across three epochs on a NVIDIA GeForce 1,080 Ti. Bold indicate best value for each category (accuracy, speedup) and dataset.

### 4.2. DVS Gesture

**Figure 2:**
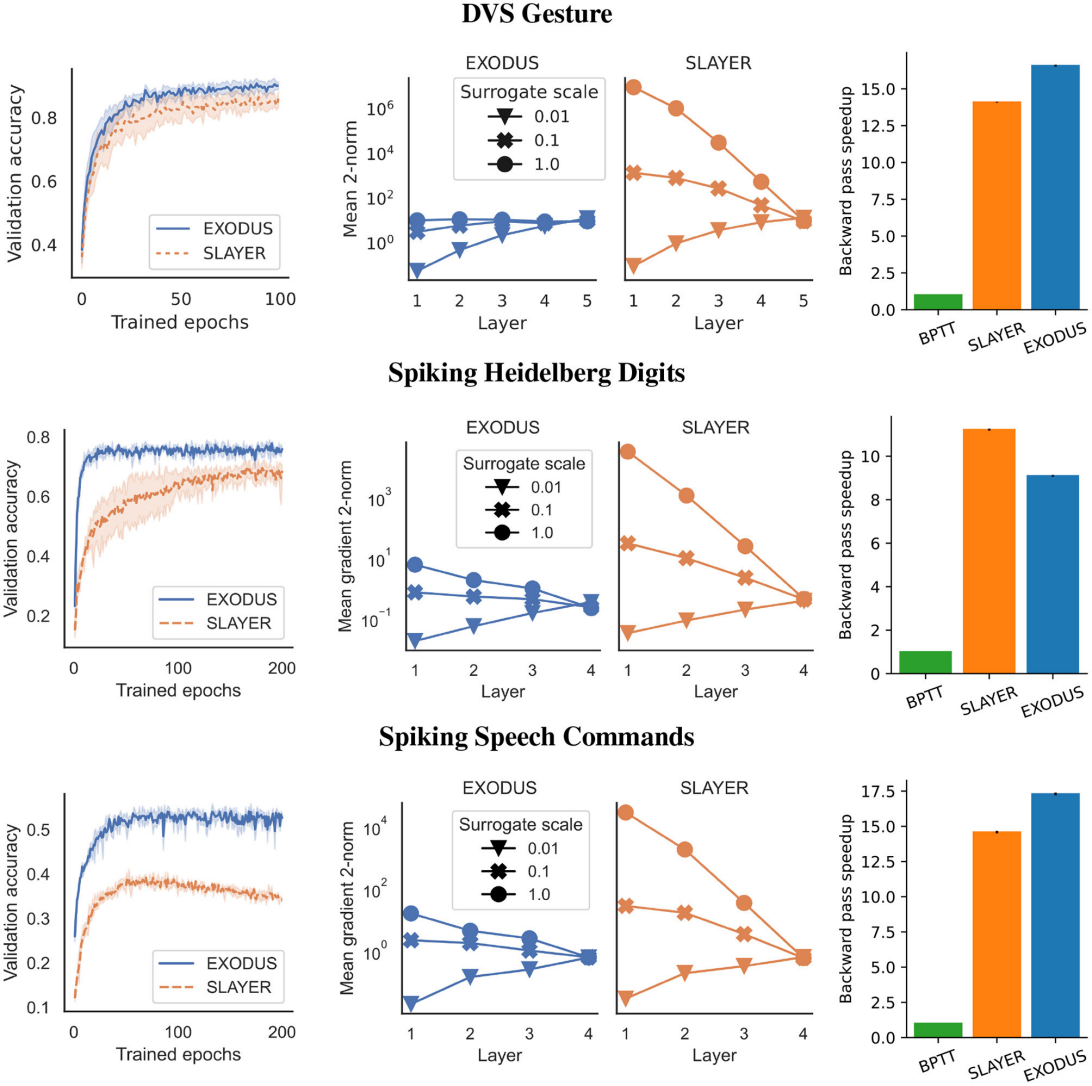
Performance on DVS Gesture **(Top row)** with 5-layer architecture, Spiking Heidelberg Digits **(Center row)** and Spiking Speech Commands **(Bottom row)** datasets. **(Left column)** Validation accuracy averaged over three runs for each algorithm using a gradient scale of 1. EXODUS (solid blue curves) generally converges much faster and reaches a higher accuracy than SLAYER (dashed orange curves), in particular for tasks with strong temporal dependencies. **(Center column)** Mean two-norms of weight gradients during training, for individual layers and different scaling of surrogate gradient. When surrogate gradients are not down-scaled, gradients for SLAYER explode toward the input layer, whereas for EXODUS they remain mostly stable. Only for very low scaling, gradients vanish for both algorithms. **(Right column)** Backward pass speedup relative to BPTT. EXODUS is the fastest on average across the three datasets.

We also test the common classification dataset of 11 different hand and arm gestures (Amir et al., 2017). Similar to Shrestha and Orchard (2018), we only use the first 1.5 s of each sample, binned to 300 time steps. To classify the gestures, we test two different spiking convolutional architectures. The smaller version has four convolutional layers as a feature extractor, followed by a fully connected layer. The larger architecture uses seven convolutional layers as feature extractor. Both versions use Integrate-and-Fire neurons, Adam and sum-over-time cross entropy loss. The left-most columns in Figure 2 and Table 1 show validation accuracies over the course of 100 training epochs for the 5-layer architecture. EXODUS reaches a higher accuracy at 92.8% than SLAYER at 87.8%. For context, the state-of-the-art result using an optimized architecture and different data pre-processing reports 98% accuracy (She et al., 2021). The center column in Figure 2 shows how weight gradients for individual layers scale under different surrogate gradient scales. SLAYER is much more sensitive to the scale, with gradients often taking excessive values for earlier layers.

### 4.3. Spiking Heidelberg Digits

This dataset is an event-based audio classification dataset with highly temporal features, with samples recorded from a silicon cochlea (Cramer et al., 2020). We used a 4-layer fully-connected architecture with Integrate-and-Fire neurons and max-over-time loss for 250 time steps. As shown in Figure 2 and Table 1, training converges faster and reaches a significantly higher accuracy using EXODUS at 78.01% compared to SLAYER at 70.58%. Gradients are much more stable for earlier layers in EXODUS. The highest accuracy result reported in the literature for this task is 83.2% using a recurrent architecture (Cramer et al., 2020).

### 4.4. Spiking speech commands

For this task we used a neuromorphic version of Google's Speech Command dataset (Cramer et al., 2020). We use the same 4-layer fully-connected architecture with Integrate-and-Fire neurons and 250 time steps as for the SHD task. Here once again our simulation results in Figure 2 and Table 1 show that gradients across layers in EXODUS are much more stable than when using SLAYER. Validation accuracy is significantly higher in this task using EXODUS with 55.4% vs 40.1% for SLAYER averaged across 3 runs. The highest accuracy result reported in the literature is 50.9% using a recurrent architecture (Cramer et al., 2020).

### 4.5. Training speedup

The training speedup in comparison to an unoptimized implementation of BPTT is shown in the right-most column in Figure 2 and Table 1. It shows relative speedups of SLAYER and EXODUS backward passes for three datasets, averaged over three runs each. We focus on the backward pass as forward passes are mathematically equivalent for all three methods. Different computation times in the forward passes are possible due to the individual implementations of the algorithms. All speed tests are executed on a NVIDIA GeForce 1,080 Ti. Our BPTT algorithm is implemented in PyTorch 1.11 and makes use of the library's native automatic differentiation functionality and GPU backend, but otherwise does not contain any custom CUDA code, which makes it much more flexible. Across three datasets, SLAYER's backward pass is 13.3 times faster than BPTT on average, whereas EXODUS is 14.3 times faster. This shows that both algorithms achieve significant speedup in comparison to a non-optimized implementation and that taking into account the reset kernel in the backward pass in EXODUS does not hurt performance at all. The minor difference between SLAYER and EXODUS can be attributed to small differences in the CUDA code implementation. Absolute backward pass timings are provided in the Table 2 in Appendix.

## 5. Discussion

We have shown that while SLAYER enables training of spiking neural networks at high computational efficiency, it does so at the cost of omitting the effect of the neuron's reset mechanism on the gradients. With our newly proposed algorithm EXODUS, we present a modification of SLAYER that computes the same gradients as those in the original BPTT while maintaining the high computational efficiency of SLAYER.

Tuning the surrogate gradient function is important when training deeper architectures (Ledinauskas et al., 2020), which can be a costly manual process when using SLAYER. EXODUS makes training deep SNN architectures from scratch easier by providing less sensitivity to the gradient scale hyperparameter. Not only does the gradient magnitude scale in a stable manner, but also the gradient direction leads to faster convergence as shown in our experiments. To demonstrate this, we picked tasks that require both spatial and temporal depth in the computational graph.

The difference between EXODUS and SLAYER is especially noticeable for long time constants and Integrate-and-Fire neurons (which do not have any leak) in the extreme case, but also persists for shorter time constants (see Appendix 6). We argue that because of the neuron's longer memory the contribution of the neuron's reset mechanism increases, which is not taken into account in SLAYER's gradient computation.

For Integrate-and-Fire neurons, both with or without leak, the terms that ensure correct representation of the reset mechanism in the gradients with EXODUS allow for a computationally efficient implementation, resulting in similar computation speeds as SLAYER. It is possible that other GPU-accelerated spiking neural network simulators, such as Norse (Pehle and Pedersen, 2021) or Spiking Jelly (Fang et al., 2020) achieve higher computational speeds than our baseline BPTT implementation. However, due to incompatibilities in the supported neuron models, a direct comparison was not possible.

Similar to Shrestha and Orchard (2018), EXODUS works exclusively with feedforward network architectures. In recurrent architectures, dynamics of individual neurons are coupled more strongly, which complicates a parallelized implementation. The application of EXODUS to such types of SNNs might be an interesting topic for future research. We hope that our method of deriving gradients through the ift is of independent interest for devising new learning strategies in a rigorous manner when no explicit functional relation exists between two or more variables.

## Data availability statement

The original contributions presented in the study are included in the article/Supplementary material, further inquiries can be directed to the corresponding author.

## Author contributions

FB and SH developed the algorithm described in this work. GL and FB conducted and analyzed the computer simulations. All authors wrote sections of the manuscript, contributed to manuscript revision, read, and approved the submitted version.
