# Supplementary material for: EXODUS: Stable and efficient training of spiking neural networks
Source: Front Neurosci. 2023 Feb 8;17:1110444. doi: 10.3389/fnins.2023.1110444 (PMC9945199; doi:10.3389/fnins.2023.1110444)
Supplement: Supplementary file 1 [file Data_Sheet_1.pdf]

frontiers

EXODUS: Stable and Efficient Training of Spiking Neural Networks — Appendix

1 EXAMPLES FOR APPLICATION OF IMPLICIT FUNCTION THEOREM

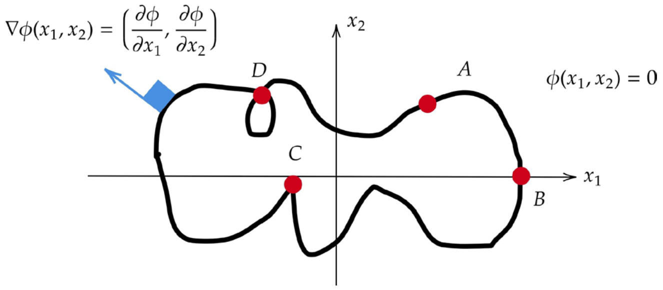

Figure S1. Illustration of the implicit function theorem.

Example 1. Fig. S1 illustrates the zero-set $\{(x_1, x_2) : \phi(x_1, x_2) = 0\}$ of a function $\phi : \mathbb{R}^2 \to \mathbb{R}$. To investigate the conditions of IFT, we first note that the gradient of ϕ denoted by $\nabla\phi = (\frac{\partial\phi}{\partial x1}, \frac{\partial\phi}{\partial x2})$ is always orthogonal to the level-set (here the zero-set) of ϕ. Thus, by observing the orthogonal vector to the level-set, we can verify if $\frac{\partial\phi}{\partial x1}$ o $\frac{\partial\phi}{\partial x2}$ are non-singular (non-zero in the scalar case we consider here).

We consider several cases:

- point D and C: at these two points the gradient vector does not exist, so the assumptions of the IFT are not fulfilled. However, we can see that at point C, one can still write x_2 as function of x_1 although this function is not differentiable.
- point A: gradient vector has nonzero vertical and horizontal components, i.e., $\frac{\partial\phi}{\partial x1} \neq 0$ and $\frac{\partial\phi}{\partial x2} \neq 0$. Thus, from IFT, one should be able to write both x_1 and x_2 locally as a differentiable function of the other.
- point B: here $\frac{\partial\phi}{\partial x1} \neq 0$ and x_1 is locally a differentiable function of x_2. However, since $\frac{\partial\phi}{\partial x2} = 0$, the assumptions of IFT do not hold for x_2. And, it is seen from Fig. S1 that x_2 cannot be written as function of x_1 in an open neighborhood of B.

One of the implications of the IFT is that, given an implicit functional relationship between a collection of variables, we are not allowed to apply partial differentiation and chain rule in an ad-hoc fashion. Otherwise, we may obtain wrong results. We illustrate this by the following example.

Example 2. (chain rule over a loopy graph) Let $\phi : \mathbb{R}^3 \to \mathbb{R}$ be a differentiable function and let (x_1, x_2, x_3) be a point in the zero-set $\phi(x_1, x_2, x_3) = 0$. By an ad-hoc application of the chain rule, one may start from $x_1 = x_1$ and apply the chain rule to obtain

$$1 = \frac{\partial x_1}{\partial x_1} = \frac{\partial x_1}{\partial x_2} \cdot \frac{\partial x_2}{\partial x_3} \cdot \frac{\partial x_3}{\partial x_1}. \tag{S1}$$

Now let us go step-by-step using the IFT and show that this result is indeed wrong. Let us consider computation of $\frac{\partial x1}{\partial x2}$. This computation simply implies that x_1 and x_2 should be treated as dependent and

independent variables, respectively. Since we have only a single equation $\phi(x_1, x_2, x_3) = 0$, we can solve one of the variables as a function of the remaining two variables. So, overall, we need to treat x_1 and (x_2, x_3) as dependent and independent variables, respectively. Now we can apply the IFT to obtain

$$\frac{\partial \phi}{\partial x_1} \cdot \frac{\partial x_1}{\partial x_2} + \frac{\partial \phi}{\partial x_2} = 0, \tag{S2}$$

which implies that

$$\frac{\partial x_1}{\partial x_2} = -\frac{\phi_2}{\phi_1}, \tag{S3}$$

where we defined the short-hand notation $\phi_i = \frac{\partial \phi}{\partial x_i}$, where we assumed that all the partial derivatives are evaluated at the point (x_1, x_2, x_3), and where we assumed that all the partial derivative ϕ_i are non-zero.

Applying the symmetry, we therefore obtain that

$$\frac{\partial x_1}{\partial x_2} \cdot \frac{\partial x_2}{\partial x_3} \cdot \frac{\partial x_3}{\partial x_1} = -\frac{\phi_2}{\phi_1} \cdot -\frac{\phi_3}{\phi_2} \cdot -\frac{\phi_1}{\phi_3} = -1. \tag{S4}$$

Comparing this with (S1) simply shows that ad-hoc application of the chain rule, especially when there is a *loopy structure* as in $x_1 \to x_2 \to x_3 \to x_1$, yields a wrong result.

2 DETAILED DERIVATION OF GRADIENTS

In the following, we provide a detailed step-by-step derivation of the the derivatives $\frac{\partial \mathbf{s}^{(l)}[n]}{\partial \mathbf{z}^{(l)}[m]}$ as defined in the main document in Section 3. Since the derivation is similar for different layers, for simplicity, we drop the superscript $^{(l)}$ here.

Let us vectorize the set of equations at a specific layer as $\boldsymbol{\phi} = [\boldsymbol{\phi}_s, \boldsymbol{\phi}_u]^\top$ where $\boldsymbol{\phi}_u = \{\boldsymbol{\phi}_u[n] : n \in [T]\}$ and $\boldsymbol{\phi}_s = \{\boldsymbol{\phi}_s[n] : n \in [T]\}$. Similarly let us vectorize the dependent variables as $\boldsymbol{\psi} = [\boldsymbol{\psi}_u, \boldsymbol{\psi}_s]^\top$ where $\boldsymbol{\psi}_u = \{\mathbf{u}[n] : n \in [T]\}$ and $\boldsymbol{\psi}_s = \{\mathbf{s}[n] : n \in [T]\}$.

To verify the conditions of Implicit Function Theorem (IFT), we can check that all the equations are differentiable functions of all the variables, provided that f_s is a differentiable function. In addition, we need to verify that the Jacobian matrix $\mathbf{J}^{\Psi}$ of the partial derivatives of $\boldsymbol{\phi}$ with respect to $\boldsymbol{\psi}$ is non-singular.

We can write it as:

$$\mathbf{J}^{\Psi} = \frac{\partial \boldsymbol{\phi}}{\partial \boldsymbol{\psi}} = \begin{pmatrix} \frac{\partial \boldsymbol{\phi_s}}{\partial \boldsymbol{s}} & \frac{\partial \boldsymbol{\phi_s}}{\partial \boldsymbol{u}} \\ \frac{\partial \boldsymbol{\phi_u}}{\partial \boldsymbol{s}} & \frac{\partial \boldsymbol{\phi_u}}{\partial \boldsymbol{u}} \end{pmatrix} = \begin{pmatrix} \boldsymbol{I}_{N_l T} & -\mathbf{f}' \\ \boldsymbol{\nu}_{-1} & \boldsymbol{I}_{N_l T} \end{pmatrix},$$

where $\boldsymbol{I}_{N_l T}$ denotes the identity matrix of order $N_l T$, and where we define

$$\mathbf{f}' := \begin{pmatrix} \boldsymbol{f}'[1] & 0 & \dots & 0 \\ 0 & \boldsymbol{f}'[2] & \dots & 0 \\ \vdots & & \ddots & \vdots \\ 0 & 0 & \dots & \boldsymbol{f}'[T \end{pmatrix},$$

with $\boldsymbol{f}'[n]$ denoting the diagonal matrix of the surrogate gradients $\{f_s'(u_i[n]) : i \in [N_l]\}$ at time instant n, and where we define

$$\boldsymbol{\nu}_{-1} = \begin{pmatrix} 0 & 0 & \dots & 0 \\ -\nu_0 \boldsymbol{I}_{N_l} & 0 & \dots & \\ \vdots & & \ddots & \vdots \\ -\nu_{T-2}\boldsymbol{I}_{N_l} & -\nu_{T-3}\boldsymbol{I}_{N_l} & \dots & 0 \end{pmatrix}.$$

Since applying simple row-wise operations will not change the invertibility of $\mathbf{J}^{\boldsymbol{\psi}}$, we add a $\boldsymbol{f}'$ multiple of the second row-block to the first row-block and obtain

$$\tilde{\mathbf{J}}^{\boldsymbol{\psi}} = \begin{pmatrix} \boldsymbol{I}_{N_lT} + \mathbf{f}'\boldsymbol{\nu}_{-1} & 0 \\ \boldsymbol{\nu}_{-1} & \boldsymbol{I}_{N_lT} \end{pmatrix}.$$

Because $\boldsymbol{\nu}_{-1}$ is a lower triangular matrix with 0-diagonal and $\mathbf{f}'$ is a diagonal matrix, $\mathbf{f}'\boldsymbol{\nu}_{-1}$ will be a lower triangular matrix with 0-diagonal. This implies that $\tilde{\mathbf{J}}^{\boldsymbol{\psi}}$ is also lower triangular with all diagonal entries equal to 1. The determinant of such a matrix is 1, which proves the invertibility of $\mathbf{J}^{\boldsymbol{\psi}}$ and verifies the non-singularity condition needed in IFT.

Since the conditions of IFT are fulfilled, we know that all the dependent variables $\boldsymbol{\psi}$ are differentiable w.r.t. independent variables $\boldsymbol{x} = \{\mathbf{z}[n] : n \in [T]\}$.

We find the derivatives $\mathbf{G} = \frac{\partial \boldsymbol{\psi}}{\partial \boldsymbol{x}}$ by solving

$$\mathbf{J}^{\boldsymbol{\psi}} \cdot \mathbf{G} = -\mathbf{J}^{\boldsymbol{x}},$$

where the Jacobian w.r.t. the independent variables is

$$\mathbf{J}^{\boldsymbol{x}} = \frac{\partial \boldsymbol{\phi}}{\partial \boldsymbol{x}} = \begin{pmatrix} \frac{\partial \boldsymbol{\phi_s}}{\partial \boldsymbol{z}} \\ \frac{\partial \boldsymbol{\phi_u}}{\partial \boldsymbol{z}} \end{pmatrix} = \begin{pmatrix} 0 \\ -\boldsymbol{I}_{N_lT} \end{pmatrix}.$$

Applying the same row-wise operations to $\mathbf{J}^{\boldsymbol{x}}$ as to $\mathbf{J}^{\boldsymbol{\psi}}$, we get

$$\tilde{\mathbf{J}}^{\boldsymbol{x}} = \begin{pmatrix} -\mathbf{f}' \\ -\boldsymbol{I}_{N_lT} \end{pmatrix}.$$

To find $\mathbf{G}$, we can now solve:

$$\tilde{\mathbf{J}}^{\boldsymbol{\psi}} \cdot \mathbf{G} = -\tilde{\mathbf{J}}^{\boldsymbol{x}}$$

$$\Leftrightarrow \begin{pmatrix} \boldsymbol{I}_{N_lT} - \mathbf{f}'\boldsymbol{\nu}_{-1} & 0 \\ \boldsymbol{\nu}_{-1} & \boldsymbol{I}_{N_lT} \end{pmatrix} \cdot \begin{pmatrix} \frac{\partial \boldsymbol{s}}{\partial \boldsymbol{z}} \\ \frac{\partial \boldsymbol{u}}{\partial \boldsymbol{z}} \end{pmatrix} = \begin{pmatrix} \mathbf{f}' \\ \boldsymbol{I}_{N_lT} \end{pmatrix}.$$

Specifically, for $\frac{\partial \boldsymbol{s}}{\partial \boldsymbol{z}}$, we find,

$$\left(\boldsymbol{I}_{N_lT} - \mathbf{f}' \cdot \boldsymbol{\nu}_{-1}\right) \cdot \frac{\partial \boldsymbol{s}}{\partial \boldsymbol{z}} = \mathbf{f}'. \tag{S5}$$

Because $\boldsymbol{I}_{N_T} + \mathbf{f}' \cdot \boldsymbol{\nu}_{-1}$ has lower triangular shape, we can solve equation (S5) through forward substitution, yielding for any $m, n \in [T]$

$$\frac{\partial \boldsymbol{s}[n]}{\partial \boldsymbol{z}[m]} - \mathbf{f}'[n] \sum_{k=1}^{n-1} \nu_{n-1-k} \frac{\partial \boldsymbol{s}[k]}{\partial \boldsymbol{z}[m]} = \delta_{m,n} \mathbf{f}'[n] \quad \Rightarrow \quad \frac{\partial \boldsymbol{s}[n]}{\partial \boldsymbol{z}[m]} = \delta_{m,n} \mathbf{f}'[n] + \mathbf{f}'[n] \sum_{k=1}^{n-1} \nu_{n-1-k} \frac{\partial \boldsymbol{s}[k]}{\partial \boldsymbol{z}[m]}. \tag{S6}$$

Starting with $n = 1$ and applying induction on n and m, and using the right-hand-side expression for $\frac{\partial \boldsymbol{s}[n]}{\partial \boldsymbol{z}[m]}$ in (S6), it is not difficult to verify that

$$\frac{\partial \boldsymbol{s}[n]}{\partial \boldsymbol{z}[m]} = 0, \quad \text{for } n < m. \tag{S7}$$

Using this result and replacing $n = m$ in (S6) yields

$$\frac{\partial \boldsymbol{s}[m]}{\partial \boldsymbol{z}[m]} = \mathbf{f}'[m]. \tag{S8}$$

Finally, inserting (S7) and (S8) into (S6), we obtain for $n > m$

$$\frac{\partial \boldsymbol{s}[n]}{\partial \boldsymbol{z}[m]} = \mathbf{f}'[n] \cdot \sum_{k=m}^{n-1} \nu_{n-1-k} \frac{\partial \boldsymbol{s}[k]}{\partial \boldsymbol{z}[m]} = \mathbf{f}'[n] \sum_{k=m}^{n-1} \nu_{n-1-k} \frac{\partial \boldsymbol{s}[k]}{\partial \boldsymbol{z}[m]}.$$

Introducing the short-hand notation $\boldsymbol{\sigma}_m[n] := \frac{\partial \boldsymbol{s}[n]}{\partial \boldsymbol{z}[m]}$ and rewriting the sum as a convolution operation, we conclude:

$$\boldsymbol{\sigma}_m[n] = \begin{cases} 0 & n < m \\ \boldsymbol{f}'[n] & n = m \\ \boldsymbol{f}'[n] \cdot \left(\boldsymbol{\nu} * \boldsymbol{\sigma}_m\right)[n-1] & n > m. \end{cases} \tag{S9}$$

■

3 GRADIENTS FOR LEAKY INTEGRATE-AND-FIRE MODEL

The Leaky Integrate-and-Fire (LIF) neuron model can be seen as a special case of the Spike Response Model (SRM), with the spike response and reset kernels ϵ and ν given as[1]

$$\epsilon_n = \alpha^n \boldsymbol{I}_{\{n \geq 0\}} \tag{S10}$$

$$\nu_n = -\alpha^n \theta \boldsymbol{I}_{\{n \geq 0\}}, \tag{S11}$$

[1] Note that the spike response kernel ϵ given here corresponds to the case where neural dynamics are modeled fully as exponential membrane decay. Synaptic dynamics could be included by replacing ϵ with $\tilde{\epsilon} := \epsilon * \rho$, with ρ being the synaptic impulse response. The validity of the following analysis would not be affected.

which allows us to find a slightly simpler formulation for $\boldsymbol{\sigma}^{(l)}_m[n] := \frac{\partial \boldsymbol{s}^{(l)}[n]}{\partial \boldsymbol{z}^{(l)}[m]}$. Dropping the superscript $^{(l)}$, the derivatives in their general form are

$$\boldsymbol{\sigma}_m[n] = \begin{cases} 0 & n < m \\ \boldsymbol{f}'[n] & n = m \\ \boldsymbol{f}'[n]\big(\boldsymbol{v} * \boldsymbol{\sigma}_m\big)[n-1] & n > m. \end{cases}$$

Let us introduce a new variable

$$\boldsymbol{\gamma}_m[n] := \begin{cases} 0 & n < m \\ \boldsymbol{I} & n = m \\ \big(\boldsymbol{v} * \boldsymbol{\sigma}_m\big)[n-1] & n > m, \end{cases} \tag{S12}$$

such that $\boldsymbol{\sigma}_m[n] = \boldsymbol{f}'[n]\boldsymbol{\gamma}_m[n]$. We first prove the following proposition.

PROPOSITION 3.1. *With v as in equation* (S11) *and for $m \geq 1$, $n > m + 1$,*

$$\boldsymbol{\gamma}_m[n] = -\theta \boldsymbol{f}'[m] \prod_{k=m+1}^{n-1} (\alpha \boldsymbol{I} - \theta \boldsymbol{f}'[k]). \tag{S13}$$

PROOF. We prove this by applying induction on n and m. It is helpful to note that for $n > m$, $\boldsymbol{\gamma}_m[n]$ can be written as

$$\boldsymbol{\gamma}_m[n] = \sum_{k=m}^{n-1} v_{n-1-k}\boldsymbol{\sigma}_m[k] = -\theta \sum_{k=m}^{n-1} \alpha^{n-1-k}\boldsymbol{\sigma}_m[k]. \tag{S14}$$

Let us consider any $m \geq 1$ and let us assume for now that there exists an $n > m + 1$ for which the proposition holds. Then for $n + 1$ we find from (S14) that

$$\begin{aligned}
\boldsymbol{\gamma}_m[n+1] &= -\theta \sum_{k=m}^{n} \alpha^{n-k}\boldsymbol{\sigma}_m[k] \\
&= -\Big(\theta\boldsymbol{\sigma}_m[n] + \theta\alpha \sum_{k=m}^{n-1} \alpha^{n-1-k}\boldsymbol{\sigma}_m[k]\Big) \\
&= -\big(\theta\boldsymbol{\gamma}_m[n]\boldsymbol{f}'[n] - \alpha\boldsymbol{\gamma}_m[n]\big) \\
&= -\boldsymbol{\gamma}_m[n]\big(\theta\boldsymbol{f}'[n] - \alpha\boldsymbol{I}\big) \\
&\overset{(i)}{=} -\boldsymbol{f}'[m]\Big(\theta \prod_{k=m+1}^{n-1}\big(\alpha\boldsymbol{I} - \theta\boldsymbol{f}'[k]\big)\Big) \cdot \big(\alpha\boldsymbol{I} - \theta\boldsymbol{f}'[n]\big) \\
&= -\theta\boldsymbol{f}'[m] \prod_{k=m+1}^{n} (\alpha\boldsymbol{I} - \theta\boldsymbol{f}'[k]),
\end{aligned}$$

where in (i), we applied the induction hypothesis for the given n and m. Up to now, we have shown that if the result is true for a given n and m with $n > m + 1$, it is true for all $\tilde{n} \geq n$. To complete the induction, therefore, we need to extend the result to $n = m + 1$. We verify this directly.

By definition of $\boldsymbol{\gamma}$, for $n = m + 1$, we have that $\boldsymbol{\gamma}_m[m+1] = -\theta\alpha^0\boldsymbol{\sigma}_m[m] = -\theta\boldsymbol{f}'[m]$ and therefore $\boldsymbol{\sigma}_m[m+1] = -\theta\boldsymbol{f}'[m]\boldsymbol{f}'[m+1]$. We can then show that for $n = m + 2$ the proposition is true:

$$\begin{aligned}\boldsymbol{\gamma}_m[m+2] &= -\theta\alpha^1\boldsymbol{\sigma}_m[m] - \theta\alpha^0\boldsymbol{\sigma}_m[m+1]\\ &= -\theta\left(\alpha\boldsymbol{f}'[m] - \theta\boldsymbol{f}'[m]\boldsymbol{f}'[m+1]\right)\\ &= -\theta\boldsymbol{f}'[m]\left(\alpha\boldsymbol{I} - \theta\boldsymbol{f}'[m+1]\right).\end{aligned}$$

Hence the proposition holds for $n = m + 2$ and therefore for all $n \geq m + 2$, independent of the choice of m. ■

With $\boldsymbol{\sigma}_m[n] = \boldsymbol{f}'[n]\boldsymbol{\gamma}_m[n]$ we can therefore write $\boldsymbol{\sigma}$ as:

$$\boldsymbol{\sigma}_m[n] = \begin{cases} 0 & n < m \\ \boldsymbol{f}'[m] & n = \\ m - \theta\boldsymbol{f}'[m]\boldsymbol{f}'[m+1] & n = m + 1 \\ -\theta\boldsymbol{f}'[m]\boldsymbol{f}'[n]\prod_{k=m+1}^{n-1}\left(\alpha\boldsymbol{I} - \theta\boldsymbol{f}'[k]\right) & n > m + \end{cases} \tag{S15}$$

3.1 Computational efficiency

We will now show that LIF neuron dynamics allow for an efficient computation of the gradients $\boldsymbol{d}^{(l)}[n] := \frac{\partial \mathcal{L}}{\partial \boldsymbol{a}^{(l)}[n]}$. Dropping the superscript $^{(l)}$, the derivatives in their general form are

$$\boldsymbol{d}[n] = \sum_{m=n}\sum_{k=m}^{T}\boldsymbol{e}[k]\boldsymbol{\sigma}_m[k]\epsilon_{m-n}. \tag{S16}$$

We rewrite Eq. (S16) in terms of $\boldsymbol{\gamma}$, as defined in Eq. (S12), keeping in minde that $\boldsymbol{\sigma}_n[k] = \boldsymbol{f}'[k]\boldsymbol{\gamma}_n[k]$ for all $k \geq n$. We furthermore insert the definition of ϵ for LIF neurons from Eq. (S10) and change the order of summation:

$$\boldsymbol{d}[n] = \sum_{m=n}\sum_{k=m}\boldsymbol{e}[k]\alpha^{m-n}\boldsymbol{f}'[k]\boldsymbol{\gamma}_m[k] = \sum_{k=n}^{T}\boldsymbol{e}[k]\boldsymbol{f}'[k]\left(\sum_{m=n}^{k}\alpha^{m-n}\boldsymbol{\gamma}_m[k]\right). \tag{S17}$$

Let us introduce another variable $\boldsymbol{\zeta}_n[k]$, defined for $n \in [T],\ l \in [L]$ and $k \geq n$, as

$$\boldsymbol{\zeta}_n[k] := \sum_{m=n}^{k}\alpha^{m-n}\boldsymbol{\gamma}_m[k], \tag{S18}$$

which allows us to write Eq. (S17) more compactly:

$$\boldsymbol{d}[n] = \sum_{k=n}^{T}\boldsymbol{e}[k]\boldsymbol{f}'[k]\boldsymbol{\zeta}_n[k]. \tag{S19}$$

We can find an expression for $\boldsymbol{\zeta}$ that is easy to compute as follows:

PROPOSITION 3.2. *With $\boldsymbol{\sigma}$, $\boldsymbol{\gamma}$, and $\boldsymbol{\zeta}$ as defined above, $\alpha > 0$, and for $n \geq 1$, $k \geq n$,*

$$\boldsymbol{\zeta}_n[k] = \begin{cases} \boldsymbol{I} & k = n \\ \prod_{m=n}^{k-1} (\alpha\boldsymbol{I} - \theta\boldsymbol{f}'[m]) & k > n. \end{cases}$$

Equivalently, $\boldsymbol{\zeta}$ can be expressed recursively as $\boldsymbol{\zeta}_n[n] = \boldsymbol{I}$ and $\boldsymbol{\zeta}_n[k+1] = \boldsymbol{\zeta}_n[k]\left(\alpha\boldsymbol{I} - \theta\boldsymbol{f}'[k]\right)$.

PROOF. For $k = n$ the proposition follows from the definitions of $\boldsymbol{\gamma}$ (S12) and $\boldsymbol{\zeta}$ (S18):

$$\boldsymbol{\zeta}_n[n] = \alpha^{n-n}\boldsymbol{\gamma}_n[n] = \boldsymbol{I}.$$

For $k > n$ first note that $\boldsymbol{\zeta}_n[k]$ can be rewritten as:

$$\begin{aligned} \boldsymbol{\zeta}_n[k] &= \alpha^{k-n}\boldsymbol{I} + \sum_{m=n}^{k-1} \alpha^{m-n}\boldsymbol{\gamma}_m[k] = \alpha^{k-n}\boldsymbol{I} + \sum_{m=n}^{k-1} \alpha^{m-n} \left(\boldsymbol{\nu} * \boldsymbol{\sigma}_m\right)[k-1] \\ &= \alpha^{k-n}\boldsymbol{I} + \sum_{m=n}^{k-1} \alpha^{m-n} \left(\sum_{r=m}^{k-1} - \theta\alpha^{k-1-r}\boldsymbol{f}'[r]\boldsymbol{\gamma}_m[r] \right), \end{aligned} \tag{S20}$$

where we inserted the definition of $\boldsymbol{\gamma}$ (S12), wrote out the convolution operation as a sum and inserted the definition of $\boldsymbol{\nu}$ (S11). Applying similar considerations to $\boldsymbol{\zeta}_n[k+1]$, we get:

$$\begin{aligned} \boldsymbol{\zeta}_n[k+1] &= \alpha^{k+1-n}\boldsymbol{I} + \sum_{m=n}^{k} \alpha^{m-n} \left(\sum_{r=m}^{k} - \theta\alpha^{k-r}\boldsymbol{f}'[r]\boldsymbol{\gamma}_m[r] \right) \\ &= \alpha^{k+1-n}\boldsymbol{I} + \sum_{m=n}^{k-1} \alpha^{m-n} \left(\sum_{r=m}^{k} - \theta\alpha^{k-r}\boldsymbol{f}'[r]\boldsymbol{\gamma}_m[r] \right) - \theta\alpha^{k-n}\boldsymbol{f}'[k]\boldsymbol{\gamma}_k[k] \\ &= \alpha^{k+1-n}\boldsymbol{I} + \sum_{m=n}^{k-1} \alpha^{m-n} \left(\alpha \left(\sum_{r=m}^{k-1} - \theta\alpha^{k-1-r}\boldsymbol{f}'[r]\boldsymbol{\gamma}_m[r] \right) - \theta\alpha^{k-k}\boldsymbol{f}'[k]\boldsymbol{\gamma}_m[k] \right) \\ &\quad - \theta\alpha^{k-n}\boldsymbol{f}'[k]\boldsymbol{\gamma}_k[k] \\ &= \alpha \left(\alpha^{k-n}\boldsymbol{I} + \sum_{m=n}^{k-1} \alpha^{m-n} \left(\sum_{r=m}^{k-1} - \theta\alpha^{k-1-r}\boldsymbol{f}'[r]\boldsymbol{\gamma}_m[r] \right) \right) - \theta\boldsymbol{f}'[k] \sum_{m=n}^{k} \alpha^{m-n}\boldsymbol{\gamma}_m[k] \\ &= \alpha\boldsymbol{\zeta}_n[k] - \theta\boldsymbol{f}'[k]\boldsymbol{\zeta}_n[k] = \boldsymbol{\zeta}_n[k]\left(\alpha\boldsymbol{I} - \theta\boldsymbol{f}'[k]\right). \end{aligned} \tag{S21}$$

For the last equality we used Eq. (S20) as well as the definition of $\boldsymbol{\zeta}$ (S18). Also note that $\boldsymbol{f}'$, and therefore also $\boldsymbol{\gamma}$ and $\boldsymbol{\zeta}$, are diagonal and thus commute. The recursive expression, as well as the equivalent closed-from in the proposition follow directly. ■

The gradient term in Equation (S19) can therefore be computed in $\mathcal{O}(T)$ time, by calculating $\boldsymbol{\zeta}_n[k]$ recursively and obtaining each summand by multiplying with $\mathbf{e}^{(l)}[k]\boldsymbol{f}'[k]$.

4 TRAINING PARAMETERS

We list the parameters that we trained our networks with. In architecture, first element is the input dimensions for one time step, a layer of 16c5 is a convolutional layer with 16 channels and a kernel size of 5 and a layer of 10l would be a simple linear layer with 10 output features. Between all weight layers we employ IF spiking layers to generate non-linear output.

Table S1. Training parameters for classification tasks.

	CIFAR10-DVS	DVS Gesture	HSD	SSC
sequence length	8	300	250	250
architecture	Wide-7B-Net as in Fang et al. (2021)	64x64x2-2c3-2p-4c3-2p-8c3-2p-16c3-11l	100-128l-128l-20l	100-128l-128l-35l
optimiser	ADAM	ADAM	ADAM	ADAM
loss	sum over time CE	sum over time CE	max over time CE	max over time CE
learning rate	1e-3	1e-3	1e-3	1e-3
mini-batch size	16	32	128	128
epochs	100	100	200	200

5 SIMILARITY TO BPTT GRADIENTS

To illustrate numerically the equivalence of the gradients computed by EXODUS to those obtained from BPTT, we measure the cosine similarity of the gradients after the first iteration when training on the Spiking Speech Commands dataset (see Section 4.4 in the main document). It is defined as

$$D = \frac{\boldsymbol{g} \cdot \boldsymbol{h}}{||\boldsymbol{g}||\ ||\boldsymbol{h}||}$$

and can be interpreted as the cosine of the angle between the two gradient vectors. Here, $\boldsymbol{g}$ and $\boldsymbol{h}$ are two gradient vectors, $\cdot$ denotes the Euclidean dot product, and $||.||$ the Euclidian norm. The cosine similarity is between -1 (vectors point in opposite directions) and 1 (vectors point in same direction). The left plot of Figure S2 shows the similarities to corresponding gradients from BPTT, for the different layers of the model. For the output layer the similarity to BPTT is 1.0 for both EXODUS and SLAYER because the gradients have not yet been propagated back through any spiking layer. For layers further down the backward pass, the similarity of SLAYER to BPTT drops down to 0.88 corresponding to an angle between the gradient vectors of about 28°. In contrast, for EXODUS it remains 1.0.

While the cosine similarity compares the direction of gradient vectors, we compare the lengths by examining the Euclidean norms. The right hand side of Figure S2 shows the lengths of the EXODUS and SLAYER gradient vectors normalized by the lengths of the corresponding BPTT gradients. Similar to the direction, for EXODUS gradients always have the same length as in BPTT. For SLAYER, on the other hand, the lengths match only for the output layer. After that they grow exponentially in comparison to those from BPTT. This highlights that EXODUS does indeed compute the same gradients as BPTT, which is not the case for SLAYER.

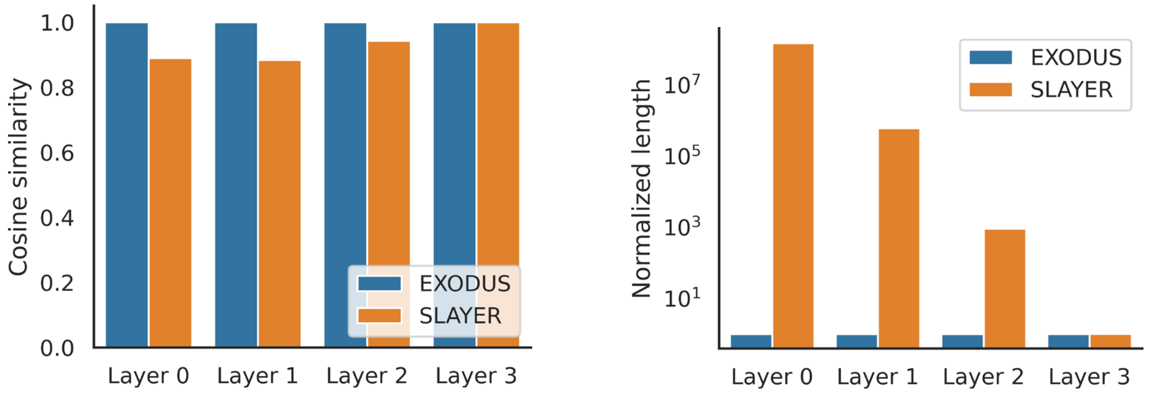

Figure S2. Comparison of gradients with from EXODUS and SLAYER to corresponding gradients from BPTT, for different layers in the network, after first iteration of training on Spiking Speech Commands dataset. **Left:** Cosine similarity. While for EXODUS the cosine similarity is 1 for all layers, for SLAYER it decreases towards the input. **Right:** Lengths of gradient vectors normalized to BPTT gradient lengths. For all EXODUS gradients the length is the same as with BPTT. For SLAYER the length is the same in the output layer and then increases exponentially.

6 POISSON SPIKE TRAIN FITTING

Similarly as in Shrestha and Orchard (2018), we generate a 250-dimensional input Poisson spike train across 200 ms as well as a target spike train for a single output neuron with 4 spikes at random times. We feed it to a single hidden layer with 25 LIF neurons, which in turn connects to a single LIF output neuron. We use mean squared error loss and the ADAM optimiser, as it is invariant to different scaling factors of the gradient. We study convergence speeds for different parameters. Example output and target spike trains can be seen in Figure S3 on top. The same figure also shows loss curves over 3000 epochs. Whereas EXODUS converges around epoch 1850 in this example, SLAYER fails to do so. We repeat our experiment for different parameters, including the time constant of the membrane potential of our LIF neurons and the learning rate. We average the loss over 5 runs for each method with different input and target spike trains and then sum up the averaged loss. This gives us an idea of speed of convergence. Figure S3 shows such summed losses for different parameters. In all cases, calculating gradients using EXODUS results in lower losses on average.

7 GRADIENTS WHEN OUTPUT IS NOT FILTERED WITH SPIKE RESPONSE

$$\boldsymbol{d}^{(L)}[n] = \frac{\partial \mathcal{L}}{\partial \boldsymbol{a}^{(L)}[n]} = \sum_{m=n}^{T}\sum_{k=m}^{T} \frac{\partial \mathcal{L}}{\partial \boldsymbol{s}^{(L)}[k]} \frac{\partial \boldsymbol{s}^{(L)}[k]}{\partial \boldsymbol{z}^{(L)}[m]} \frac{\partial \boldsymbol{z}^{(L)}[m]}{\partial \boldsymbol{a}^{(L)}[n]} = \sum_{k=m}^{T}\sum_{m=n}^{T} \frac{\partial \mathcal{L}}{\partial \boldsymbol{s}^{(L)}[k]} \boldsymbol{\sigma}_m^{(L)}[k]\epsilon_{m-n}.$$

The term $\boldsymbol{e}^{(L)}$ disappears. For $l < L$, $\boldsymbol{d}^{(l)}$ and $\boldsymbol{e}^{(l)}$ are defined the same way as before.

8 TRAINING TIME RAW NUMBERS

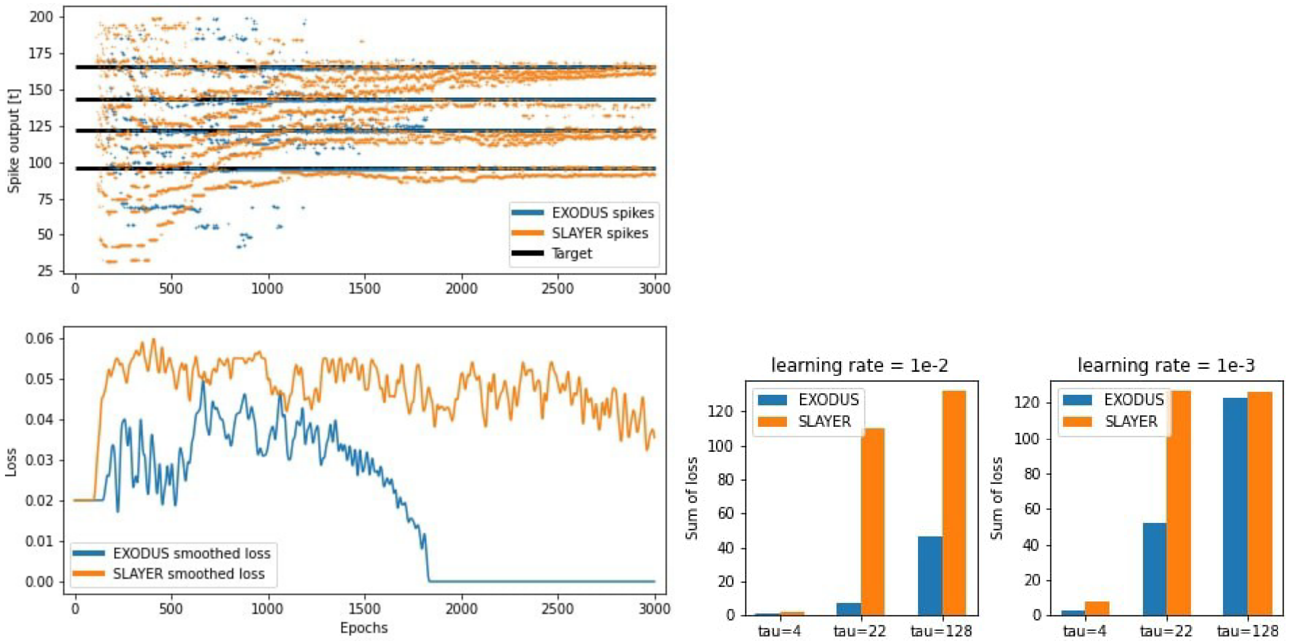

Figure S3. Poisson spike train fitting. **Upper:** spike output for networks when trained with EXODUS and SLAYER as well as target spike train, all tracked across epochs. **Lower:** Loss over time for example experiment. EXODUS shows faster convergence than SLAYER. **Right:** Sum of averaged loss across 5 experiments for one parameter combination (learning rate, LIF time constant tau) as well as method EXODUS/SLAYER.

	Time per training step [ms]					
	EXODUS		SLAYER		BPTT	
	forward	backward	forward	backward	forward	backward
DVS	**147.47** ± **0.4**	**89.63** ± **0.3**	246.13 ± 0.7	105.41 ± 0.2	970.72 ± 861.2	1485.28 ± 1.1
HSD	**14.84** ± **0.0**	26.24 ± 0.1	64.48 ± 0.3	**21.26** ± **0.0**	334.74 ± 180.5	238.63 ± 0.9
SSC	**4.60** ± **0.0**	**9.60** ± **0.0**	36.54 ± 0.2	11.38 ± 0.0	123.29 ± 10.6	166.08 ± 0.8

Table S2. Training time is measured in ms per training step, on a NVIDIA GeForce 1080 Ti averaged across 3 epochs.
